# Recent Advances in d-Lactic Acid Production from Renewable Resources: Case Studies on Agro-Industrial Waste Streams

**DOI:** 10.17113/ftb.57.03.19.6023

**Published:** 2019-09

**Authors:** Maria Alexandri, Roland Schneider, Kerstin Mehlmann, Joachim Venus

**Affiliations:** Leibniz Institute for Agricultural Engineering and Bioeconomy, Department of Bioengineering, Max-Eyth Allee 100, 14469 Potsdam, Germany

**Keywords:** d-lactic acid, renewable resources, polylactic acid, microbial fermentations, downstream

## Abstract

The production of biodegradable polymers as alternatives to petroleum-based plastics has gained significant attention in the past years. To this end, polylactic acid (PLA) constitutes a promising alternative, finding various applications from food packaging to pharmaceuticals. Recent studies have shown that d-lactic acid plays a vital role in the production of heat-resistant PLA. At the same time, the utilization of renewable resources is imperative in order to decrease the production cost. This review aims to provide a synopsis of the current state of the art regarding d-lactic acid production *via* fermentation, focusing on the exploitation of waste and byproduct streams. An overview of potential downstream separation schemes is also given. Additionally, three case studies are presented and discussed, reporting the obtained results utilizing acid whey, coffee mucilage and hydrolysate from rice husks as alternative feedstocks for d-lactic acid production.

## INTRODUCTION

Lactic acid is a well-established bio-based chemical, which can be produced using renewable resources by various wild-type strains. Traditionally, lactic acid is synthesized *via* the chemical hydrolysis of lactonitrile, a process that leads to the production of a racemic mixture of d(-)- and l(+)-lactic acid. In contrast to the petrochemical route, biotechnological production of lactic acid has various advantages such as high enantiomeric purity, utilization of inexpensive substrates and environmentally benign processes (*1*). The industrial production of lactic acid is mainly based on its fermentative route since it is estimated that about 90% is produced microbiologically (*1*).

Global lactic acid production is approx. 270 000 t per year, as it presents a broad range of applications in food industry, cosmetics and also in pharmaceuticals (*2*). Its market demand presents an annual growth rate of 10%, which is mainly attributed to the utilization of lactic acid for the synthesis of polylactic acid (PLA) (*3*). PLA is a biodegradable polymer used in the production of food packaging materials such as containers, wraps, single use trays, but also mulch films and garbage bags (*3*). In Europe, the demand for PLA accounts for 25 000 t per year, and it is expected to reach 650 000 t by 2025 (*3*).

Enantiomeric ratio and polymerization process are the two key parameters affecting the properties of PLA (*2*, *4*, *5*). The polymer produced from a racemic mixture of l- and d-lactic acid (PDLLA) is both thermally and mechanically unstable, also with a short shelf life. PLA produced from pure l- (PLLA) or d-lactic acid (PDLA) possesses better properties (higher melting point), but still its application is hindered due to its thermal instability. Recent research on PLA properties has shown that both thermal and mechanical stability are enhanced when an isotactic stereocomplex of PLLA and PDLA is formed (*4*). The chemical stabilization of the polymer chains is achieved *via* hydrogen and van der Waals bonding (*2*, *6*). With this technique, the melting temperature of the polymer is 230 instead of 60 °C of the PLLA (*4*).

The fermentative production of l-lactic acid of high optical purity has been extensively studied using different strains and substrates such as municipal solid wastes, lignocellulosic hydrolysates and food wastes (*7*, *8*). Its industrial production has also been reported since 1881, and many companies or pilot plants are operating on l-lactic acid production using renewable resources such as Corbion (Amsterdam, The Netherlands), Galactic (Celles, Belgium), and NatureWorks LLC (Minnetonka, MN, USA) (*1*). Among these companies, only Corbion had launched PDLA in its portfolio.

This review aims to provide an overview of the current research on d-lactic acid production from renewable resources. Additionally, three experimental case studies dealing with the valorization of acid whey, coffee mucilage and rice husks will be presented and discussed as alternative feedstocks for d-lactic acid production.

## d-LACTIC ACID PRODUCERS

Highly pure l-lactic acid can be produced by a broad range of microorganisms, such as bacteria, fungi, algae and cyanobacteria. On the other hand, most of d-lactic acid-producing microorganisms produce either the racemic mixture or other organic acids, such as acetic acid or succinic acid (*9*). For efficient fermentative production of d-lactic acid, it is crucial to identify or even engineer the adequate strain. Moreover, since the cost of the substrate plays an important role for the economic feasibility of the process, the utilized strain needs to be able to consume pentoses, which are mainly present in lignocellulosics, the emerging renewable resource.

The most well-known wild-type d-lactic acid-producing strains include *Lactobacillus delbrueckii* and *Sporolactobacillus* sp. (*9*, *10*). These microorganisms are characterized as Gram--positive, facultative anaerobic, mesophilic and catalase negative. The optimum temperatures for the growth of *Lactobacillus delbrueckii* range between 45 and 47 °C, whereas the strains belonging to *Sporolactobacillus* sp. prefer lower temperatures, in the range of 20–45 °C (*9*). Lactic acid production occurs either through homofermentative or heterofermentative pathway. Homofermentative strains, such as *L. delbrueckii*, produce only lactic acid as end-product of their metabolism, *via* the Embden-Meyerhof-Parnas pathway in the presence of glucose as carbon source (*11*). d-lactic acid production is catalyzed by the enzyme d-lactate dehydrogenase (d-LDH), and theoretically 1 g of lactic acid can be produced from 1 g of glucose (*12*). However, homofermentative strains cannot assimilate pentoses, such as xylose and arabinose, which are the main sugars present in lignocellulosic hydrolysates. Heterofermentative strains, such as *L. brevis* and *L. pentosus*, are able to consume pentoses through the phosphoketolase (PK) pathway, producing lactic acid as the main end product and acetic acid, ethanol and/or formic acid as byproducts (*13*). Therefore, the theoretical d-lactic acid yield in this case is 0.5 g/g from glucose (hexoses) and 0.6 g/g from xylose (pentoses) (*9*).

High d-lactic acid concentrations of more than 200 g/L have already been demonstrated from commercial glucose, by employing *Sporolactobacillus inulinus*. Wang *et al.* (*14*) reported the production of 207 g/L of d-lactic acid of high optical purity (99.3%) from glucose with peanut meal as nitrogen source in a fed-batch fermentation. In the recent work of Klotz *et al.* (*2*), product concentrations up to 222 g/L were achieved with *S. inulinus* DSM 20348 in a fed-batch fermentation, in which amino acids and vitamins were also added.

Recombinant strains of *Escherichia coli*, *Bacillus coagulans*, *Corynebacterium glutamicum* and *Bacillus licheniformis* have also been evaluated for d-lactic acid production. In the study of Awasthi *et al.* (*10*), a thermotolerant *B. subtilis* derivative was metabolically engineered to produce d-lactic acid *via* insertion of five heterologous *IdhA* (d-LDH) genes. The authors cloned two genes responsible for d-lactic acid from an engineered *B. coagulans* strain; an altered glycerol (polyol) dehydrogenase (*gldA101*) and *IdhA*. The results indicated that neither the insertion of the gene encoding *gldA101* nor *IdhA* had a positive effect on d-lactic acid production by the engineered strains. Three *IdhA* genes from three different *L. delbrueckii* strains were also tested, since this microorganism can grow in a wide range of temperatures. The d-LDH from *L. delbrueckii* ssp. *bulgaricus* was more stable at 50 °C than the ones from *L. delbrueckii* ssp. *lactis*, so these genes were chosen for engineering *B. subtilis*. The engineered strain produced approx. 1 M d-lactic acid in batch fermentation mode, with a yield of 0.98 g/g on consumed glucose.

An alkaliphilic *Bacillus* sp. was also engineered by deletion of the *Idh* gene and insertion of the *IdhA* gene from *L. delbrueckii*. At the same time, for enhanced d-lactic acid, the gene encoding exopolysaccharide biosynthesis (*epsD*) was disrupted. The strain produced almost 144 g/L of optically pure d--lactic acid (99.85%), with a yield of 0.96 g/g and productivity of 1.67 g/(L·h), in a fed-batch fermentation using peanut meal as nitrogen source and commercial glucose as carbon source (*15*).

Even though there are some studies reporting l-lactic acid fermentation from renewable resources on a pilot or technical scale, the information for d-lactic acid is quite limited. The most representative work so far is the publication of Liu *et al.* (*16*) that demonstrated d-lactic acid production from a genetically engineered *E. coli* strain on a pilot scale using a working volume of 3 t. Glucose was the sole carbon source together with various mineral salts, and Ca(OH)_2_ was used as neutralizing agent. Operating in batch mode, the authors obtained a final d-lactic acid value of 126 kg/t, with a yield of 0.97 g/g and productivity of 6 kg/(t·h). The optical purity of the derived lactic acid was 99.5%.

## d-LACTIC ACID PRODUCTION FROM RENEWABLE RESOURCES

The efficient production of d-lactic acid from commercial glucose has already been demonstrated in the previous section. However, the main challenge nowadays lies in developing efficient bioprocesses based on renewable resources. Table 1 (*13*, *17*-*34*) lists recent publications dealing with the utilization of alternative substrates for d-lactic acid production using wild-type or engineered strains. Lignocellulosic biomass constitutes one of the most promising substrates, since it is abundant, inexpensive, and rich in structural polysaccharides (cellulose, hemicellulose) that after pretreatment and hydrolysis can be converted to value-added products and chemicals (*7*, *35*). d-lactic acid production from lignocellulosic hydrolysates has not been extensively studied. Hama *et al.* (*18*) employed a metabolically engineered *L. plantarum* for simultaneous saccharification and fermentation (SSF) of hardwood pulp. Hardwood pulp is a byproduct of the pulp and paper industry, generated after biomass fractionation. The engineered strain *L. plantarum* 8826 *ΔldhL1*::P*xylAB*-*Δxpk1*::*tkt-Δxpk2*::P*xylAB* was able to utilize both glucose and xylose, resulting in 102.3 g/L d-lactic acid with high optical purity (99.2%), a yield on total sugars of 0.879 g/g, and a productivity of 2.29 g/(L·h).

**Table 1:** Recent studies on d-lactic acid production from renewable feedstocks using wild-type and engineered strains

Strain	Substrate	Nitrogen source/other nutrients	Fermentation mode	*γ*(d-lactic acid)/(g/L)	Optical purity/%	*Y*(d-lactic acid)/(g/g)	*r*_P_(d-lactic acid)/(g/(L·h))	Byproduct	Reference
*Lactobacillus delbrueckii* ssp. *delbrueckii* NBRC 3202	Millet bran hydrolysate	–	Batch, shake flasks	25.38	97.79	1.15*	0.26	n.m.	(*17*)
Metabolically engineered *Lactobacillus plantarum*	Hardwood pulp by mechanical milling	–	Batch	102.3	99.2	0.88	2.29	–	(*18*)
Metabolically engineered *Lactobacillus plantarum* with initial acid adaptation	Brown rice	–	Batch	117.1	99.6	0.93	0.81	–	(*19*)
*Sporolactobacillus inulinus* NBRC 13595	*Borassus flabellifer* sugars	Whey protein hydrolysate	Batch	189	–	–	5.25	–	(*20*)
*L. delbrueckii* sp. *bulgaricus*	Corn stover hydrolysate	MRS solution	Batch	18	99	–	0.41	n.m.	(*21*)
*Sporolactobacillus inulinus* YBS1-5	Corncob residue hydrolysates	Cottonseed meal hydrolysate	Fed-batch	107.2	99.2	0.85	1.19	n.m.	(*22*)
Recombinant *L. plantarum* NCIM B 8826 *ΔldhL*1-*pLEM*-*xylAB*	Corn stover hydrolysates	Soybean meal hydrolysate	Fed-batch	61.4	99	0.77	0.32	Acetic acid	(*13*)
*Sporolactobacillus inulinus* YBS1-5	Corn stover hydrolysates	Yeast extract, corn steep liquor	Batch	70.7	–	0.82	0.65	–	(*23*)
*Lactobacillus coryniformis* subsp. *torquens*	Pulp mill residue	MRS medium without glucose	Batch	57	99.1	0.97	2.8	Acetic acid	(*24*)
*L. brevis* ATCC 367 and *L. plantarum* ATCC 21028	Poplar hydrolysate	MRS without glucose	Batch (sequential cofermentation)	31.8	50	0.80	0.48	Acetic acid	(*25*)
*L. brevis* ATCC 367 and *L. plantarum* ATCC 21028	Alkali-pretreated corn stover	MRS without glucose	Batch^a^ (sequential cofermentation)	31.2	43.2	0.78	0.43	Acetic acid	(*25*)
Mixed culture of *L. rhamnosus* and *L. brevis*	Corn stover	–	Batch^a^	20.95	–	0.70	0.58	Acetic acid	(*26*)
*L. delbrueckii* ssp. *lactis* ATCC 4797	Casein whey permeate (~50 g/L lactose)	Casein hydrolysate	Batch	24.3	98.2	0.49	0.61	n.m.	(*27*)
Engineered *Pediococcus acidilactici* (*Idh* gene disruption)	Corn stover hydrolysate	MRS without glucose	Batch^a^	77.78	99.32	0.58	1.02	n.m.	(*28*)
*Sporolactobacillus inulinus* Y2-8	Corn flour hydrolysate	Yeast extract	Batch^b^	145.8	>99	0.97	1.62	–	(*29*)
*L. bulgaricus* GCMCC 1.6970	Chicory-derived inulin	MRS without glucose	Batch^a^	123.6	>99.9	0.98	1.72	–	(*30*)
*S. nakayamae*	Commercial sucrose	Peanut flour	Batch	112.93	98.8	0.98	1.57	–	(*31*)
*L. bulgaricus* CGMCC 1.6970	Whey	Whey enzymatic hydrolysate and yeast extract	Fed-batch	113.18	–	–	2.36	–	(*32*)
*L. coryniformis* ATCC 25600	Waste *Curcuma longa*	Soybean meal	Batch^a^	91.67	99.5	0.65	2.08	–	(*33*)
*L. delbrueckii* ssp. *delbrueckii*	Waste orange peels	Corn steep liquor	Batch	–	>95	0.88	2.35		(*34*)

^a^Simultaneous saccharification and fermentation, ^b^cells were immobilized in fibrous bed bioreactor, n.m.=not mentioned

Corncob residues and cottonseed meal were evaluated by Bai *et al.* (*22*) for d-lactic acid production using the strain *S. inulinus* YBS1-5. The hemicellulosic part of corncobs was utilized for the production of xylitol and furfural, leaving the cellulosic stream as a waste residue. The pretreatment required for hemicellulose exploitation renders cellulose more susceptible to enzymatic hydrolysis. Lactic acid is produced with a growth-associated mechanism, meaning that the selection of an adequate nitrogen source is also of major importance in order to achieve efficient cell growth. To this end, the authors studied four alternative nitrogen sources: cottonseed meal, soybean meal, peanut meal and corn wine dregs. Their screening revealed that among the different agro-industrial residues, cottonseed meal gave the most promising results, since it is found to be rich in proteins and vitamins. In order to facilitate the utilization of the proteins in cottonseed meal by the strain, enzymatic hydrolysis was carried out with neutral proteases. When the hydrolysates from two different streams were combined in a fed-batch fermentation, the strain produced 107 g/L lactic acid, with a yield and productivity of 0.85 g/g and 1.2 g/(L·h), respectively. The resulting d-lactic acid was of high enantiomeric purity (99.2%); however, xylose accumulation was observed.

Besides lignocellulosics, other waste or byproduct streams have been suggested as alternative carbon and/or nitrogen sources for d-lactic acid production. Reddy Tadi *et al.* (*20*) achieved high d-lactic acid concentrations (189 g/L) by combining sugars derived from *Borassus flabellifer* with whey protein hydrolysate and employing *S. inulinus* NBRC 13595. Zhao *et al.* (*29*) achieved a final concentration of 145.8 g/L from corn flour hydrolysate using *S. inulinus* Y2-8. When *S. inulinus* cells were immobilized in a fibrous bed reactor, final d-lactic acid concentration was enhanced by 37.67%.

Production of d-lactic acid from a *L. bulgaricus* strain using renewable inulin was also reported by Xu *et al.* (*30*). Inulin is a naturally occurring polysaccharide, serving as energy storage for many plants such as chicory, Jerulasem artichoke and dahlia among others, mainly found in their roots or tubers (*36*). It is a fructose polymer, having glucosyl moieties at the end-chains. Enzymatic or acidic hydrolysis of inulin leads to the production of feedstock rich in fructose and glucose. The strain performed better when operating under SSF, since fructose inhibition was prevented. Under these conditions the strain was able to produce 123.6 g/L of optically pure d-lactic acid (>99.9%).

Recently, Liu *et al*. (*32*) suggested cheese whey powder as a complete feedstock for d-lactic acid production employing *L. bulgaricus*. Neutral protease was selected for the hydrolysis of the protein content in cheese whey powder, in order to facilitate their assimilation by the microorganism. Their results indicated that the addition of a small amount of yeast extract significantly enhanced the final d-lactic acid concentration. Finally, 113.18 g/L of d-lactic acid was produced in a fed-batch fermentation, with a productivity of 2.36 g/(L·h).

Beitel *et al*. (*31*) reported a d-lactic acid concentration of 112.93 g/L using the strain *S. nakayamae* and commercial sucrose and peanut flour as carbon and nitrogen sources, respectively. The authors initially tested the effect of different inexpensive carbon sources (molasses, commercial sucrose, sugarcane juice and whey) on lactic acid production, yield and productivity. The strain grew poorly on molasses and whey, but it was able to produce high amounts of lactic acid from sucrose and sugarcane juice. Using commercial sucrose as carbon source, the authors subsequently evaluated the effect of different nitrogen sources. Among corn steep liquor, Proflo, peanut flour, soybean flour and urea, Proflo and peanut flour led to the highest lactic acid production (more than 50 g/L) and productivities of more than 1 g/(L·h). Liu *et al*. (*32*) also achieved high d-lactic acid concentration of 113.18 g/L by employing cheese whey as carbon and nitrogen source combined with 9 g/L of yeast extract.

*B. coagulans* is a very attractive l-lactic acid producer since it is able to ferment pentoses, making it an excellent candidate for fermentation of lignocellulosic hydrolysates (*14*). Moreover, its thermotolerant nature (50–55 °C) minimizes the need for sterilized conditions. This strain has already been reported for efficient l-lactic acid fermentation from renewable resources like coffee pulp (*37*), coffee mucilage (*8*) and corn stover (*38*). Deletion of both *Idh* and *alsS* (acetolactate synthase), with subsequent growth-based selection, led to the development of the mutant strain QZ19, which could accumulate 90 g/L of optically pure d-lactic acid (*14*). The strain was also tested on sorghum juice, resulting in almost 125 g/L d-lactic acid, with a productivity of 5 g/(L·h). An engineered *L. plantarum* strain was also evaluated for fermentation of corn stover and soybean meal extract to d-lactic acid. Genes encoding xylose isomerase and xylulokinase were inserted, giving the recombinant strain *L. plantarum* NCIMB 8826 *DldhL1-pLEM-xylAB*, which was able to ferment the renewable resources (containing both glucose and xylose), resulting in 61.4 g/L lactic acid, with a yield of 0.77 g/g in a fed-batch fermentation (*13*).

## CO-CULTIVATION STRATEGIES

The efficiency of upstream process strongly depends on the fermentability of the substrate. Unconsumed sugars lead to low product yields and at the same time impede the purification of lactic acid. Mixed cultures or co-cultivation (cofermentation) strategies could be employed aiming to increase the fermentability of the lignocellulose-derived substrates. This strategy is based on the utilization of a homofermentative and a heterofermentative strain either simultaneously or sequentially. The homofermentative strain converts glucose into highly pure d-lactic acid, and the heterofermentative one consumes the pentoses. This approach leads to better substrate utilization and a controlled byproduct formation from the heterofermentative strain. A mixed culture of *L. rhamnosus* and *L. brevis* was tested for the fermentation of corn stover hydrolysate, increasing the conversion of substrate to lactic acid for almost 30%, in comparison to pure cultures (*26*). Zhang and Vadlani (*25*) accomplished an enhanced fermentability of poplar and corn stover hydrolysates by sequential cofermentation of *L. brevis* and *L. plantarum*. *L. brevis* was added after 20 h of fermentation, when poplar hydrolysate was employed, leading to a 13% increased yield, but with only 50% optical purity. When corn stover was used as fermentation substrate, a simultaneous saccharification and fermentation strategy was followed. The authors utilized Cellic® CTec2 (Novozymes Inc., Franklinton, NC, USA) for sugar monomer release from the substrate. When all glucose was depleted by *L. plantarum*, *L. brevis* was added for xylose consumption. Final lactic acid concentration was 31.2 g/L, but also 6.3 g/L of acetic acid was produced, and the optical purity was only 43.2%.

## ACID TOLERANCE

The optimum pH for most lactic acid microorganisms ranges between 5.0 and 6.0, values above the pK_a_ of the acid (pK_a_=3.8). At pH values higher than the pK_a_, the acid is mainly found at its dissociated form, greatly incommoding its separation from the fermentation medium. For its efficient separation and purification, lactic acid needs to be in its free acid form, meaning that further addition of chemicals and purification steps is required (section DOWNSTREAM PROCESSING). The development of robust strains towards acidic pH would decrease the downstream costs and minimize waste generation. Genetic engineering of the strains has been proposed in order to achieve acid tolerance, since free lactic acid is highly inhibitory towards the fermenting microorganisms (*1*, *12*, *39*). The mechanism of inhibition is mainly described as increased proton transfer through the plasma membrane, causing a disturbance of intracellular pH (*40*). Decrease in intracellular pH, which is supposed to be neutral, has a linearly decreasing effect on cell propagation (*40*).

Zheng *et al*. (*41*) applied genome shuffling in *S. inulinus* ATCC 15538, aiming to enhance the strain’s tolerance to d-lactic acid, and at the same time to increase the productivity. After three rounds of genome shuffling, the mutant strain produced 119% more d-lactic acid than the wild-type in a fed-batch fermentation with glucose as carbon source, operating at pH=5. At the same time, the viability of the mutant strains at acidic pH values was enhanced, since they were able to grow at pH=4, when their optimum was pH=6.5.

Yeast strains have also been used for d-lactic acid production, due to their ability to grow at lower pH values than bacteria. Baek *et al*. (*42*) combined metabolic engineering and adaptive evolution of the strain *Saccharomyces cerevisiae* in order to induce d-lactic acid production and tolerance of glucose. A fed-batch fermentation resulted in 112 g/L of d-lactic acid in shake flasks with yield and productivity of 0.80 g/g and 2.2 g/(L·h), respectively. In a recent study of Park *et al*. (*43*), a newly isolated *Pichia kudriavzevii* strain was engineered to enhance lactic acid production and at the same time it was adapted to tolerate high acid concentrations. Another interesting feature of the strain was the fact that it was able to grow at low pH conditions. Operating in fed-batch fermentation mode with glucose as carbon source, the strain was able to produce 154 g/L of d-lactic acid at pH=4.7, while it was also possible to accumulate lactic acid up to 135 g/L at pH=3.6. The yeast strain *Kluyveromyces marxianus* was metabolically engineered by redirection of its metabolic pathway from ethanol to lactic acid (*44*). The recombinant strain could ferment Jerusalem artichoke tuber powder (inulin-rich) at pH=6, resulting in the production of 122 g/L lactic acid with an optical purity of more than 99%.

## DOWNSTREAM PROCESSING

An efficient upstream process must be coupled with a cost-efficient and environmentally benign downstream separation method. The steps required for the separation and purification of lactic acid from the fermentation broth are crucial for the final quality of the product (*9*). The cost of the downstream process can account for 50% of the costs (*9*).

In general, the same downstream process that needs to be followed for l-lactic acid must also be utilized for d-lactic acid. Currently, the industrial process for separation of lactic acid from the fermentation broth involves its precipitation with calcium hydroxide or calcium carbonate. Before precipitation, biomass is separated *via* filtration. Lactic acid is then separated from the fermentation broth as a precipitate, and it is recovered with excess of sulphuric acid. The main drawback of this process is the generation of high amounts of gypsum that are considered a waste stream (*45*). Research has been focusing on finding more environmentally friendly alternatives; however, they have been tested only on laboratory or pilot scales.

Alternative separation technologies involve reactive extraction, membrane separation or *in situ* recovery *via* solvent extraction. Chromatography using cation- or/and anion-exchange resins has been reported as efficient purification method, mainly due to its selectivity, low waste generation and operational effectiveness (*35*). Another industrially attractive process is reactive distillation, in which lactic acid is esterified to an alcohol and then the ester is hydrolysed to give free lactic acid (*46*, *47*).

Beitel *et al*. (*31*) attempted to separate and purify d-lactic acid from the fermentation broth. The medium contained 88.27 g/L of d-lactic acid and 13.5 g/L of residual sugars. Cell biomass was removed *via* centrifugation, and then the supernatant was filtered through activated carbon and Celite twice. The filtrate was subsequently treated with the cation exchange resin Amberlite IRA 120. The recovery yield of lactic acid from the fermentation broth was 79%, while 98% of the remaining sugars were removed.

## TECHNO-ECONOMIC ASSESSMENT OF d-LACTIC ACID PRODUCTION

The success of the biotechnological production of d-lactic acid from renewable resources is strongly affected by the sustainability issues as well as the cost-competiveness of the individual process parameters and operations. Environmental and socio-economic aspects should be also taken into account (*48*). The most recent studies on techno-economic analysis of l-lactic acid production from various renewable resources are summarized in the review article of Alves de Oliveira *et al.* (*1*). The use of lignocellulosic biomass requires pretreatment before fermentation. The type of pretreatment contributes significantly to the overall cost of the process as well as the environmental impact, especially when chemicals are utilized. The nitrogen source is another major cost contributor. Then, downstream process steps also require careful selection, since high purity is required with minimum use of chemicals and waste generation.

The complexity of such a process is described in the work of Gezae Daful and Görgens (*49*), in which six different scenarios for the biotechnological production of l-lactic acid from sugarcane bagasse and leaves were proposed and discussed in terms of cost efficiency and environmental impact. Biomass pretreatment was based on steam explosion, leading to the formation of a hemicellulose-rich liquid fraction and a cellulignin one. The authors decided to investigate the production of lactic acid either from C5 or C6 sugars and couple its production with other fuels and chemicals. The use of Ca(OH)_2_ or Mg(OH)_2_ (coupled with recycling of Mg(OH)_2_) or an acid-tolerant thermophilic *Bacillus coagulans* were other process parameters under investigation. Reactive distillation was the selected downstream separation process. The scenarios in which the cellulose fraction was selected as a carbon source were more profitable than hemicellulose-based ones. The selected strain, a genetically engineered *E. coli* WL204, was able to produce l-lactic acid of high purity from both glucose and xylose. This process resulted in 7–10% increase in total capital investment, 58–86% increase in operating cost and 12–18% revenue increase in comparison to the other studied scenarios. Higher environmental impact was observed when Ca(OH)_2_ and H_2_SO_4_ were utilized due to the formation of gypsum. According to that study, the production of 1 t of lactic acid is accompanied by the generation of 1 t of gypsum as solid waste. The use and recycling of Mg(OH)_2_ was more favourable both in terms of economic feasibility and environmental impact.

Sikder *et al.* (*50*) evaluated the economic feasibility of a membrane-integrated bioreactor system, in which lactic acid was produced from sugarcane juice. The individual processing steps involved sterilization, fermentation, microfiltration, nanofiltration and vacuum evaporation. Raw material and yeast extract were the two largest cost contributors, accounting for about 6 and 87% of the total operating cost, respectively. Approximately 36% of the total fixed capital cost was attributed to the fermentation step, whereas the membrane units were responsible for only 2%. Total product cost of this process was calculated at 3.15 US$/kg for 80% (*m*/*m*) concentrated lactic acid with 95% purity. The authors concluded that product cost could be further reduced if alternative and cheaper nitrogen sources were applied.

Techno-economic analyses of d-lactic acid from renewable resources are scarce in the literature. De la Torre *at al.* (*34*) calculated the cost contribution of the nitrogen source. The authors carried out fermentations to test yeast extract, malt extract and corn steep liquor. An amount of 2.64 g/L of nitrogen was found regardless of the source, which resulted in d-lactic acid yield of about 80%. However, the cost varies significantly among the different sources, with malt extract being the most expensive one. More specifically, the authors reported that when malt extract was selected as nitrogen source, the cost was up to 3482 US$/t of d-lactic acid, when its industrial price was 1300 US$/t. When using yeast extract, the cost was calculated up to 500 US$/t of d-lactic acid, while for corn steep liquor the cost was only 90.78 US$/t. The researchers concluded that even when corn steep liquor needs to be added at higher amount than malt and yeast extracts, its cost is still significantly lower than of the other two nitrogen sources.

Since nutritional requirements can be different for the d-lactic acid-producing strains, more studies of the techno-economic assessment that are more product-specific need to be carried out. Techniques like co-cultivation, which are not attractive in the case of l-lactic acid, are quite promising for d-lactic acid and this scenario should also be investigated in terms of economic viability. If wild-type strains are used, some byproducts like acetic acid and ethanol might also be co-produced, and the efficiency of the downstream separation steps must be studied as well, and subsequently these steps will also affect both the economics of the process and the environmental impact.

## CASE STUDIES

### Acid whey

Whey is the main byproduct derived from the dairy industry, and it is divided into two categories: sweet and acid whey. Sweet whey is generated from the production of cheese, whereas acid whey is the byproduct stream of acid-coagulated dairies like yoghurt (*51*). Sweet whey is processed mainly *via* spray drying to whey powder or whey protein concentrate and sold for various food applications (*51*). Acid whey is rich in lactic acid, making the powder too hygroscopic, resulting in agglomeration, which is quite problematic for spray dryers (*52*). Consequently, acid whey is either used as animal feed or discarded, causing serious environmental problems. The worldwide production of fresh dairy products in 2016 was more than 400 million tonnes, of which almost 80 million tonnes were in Europe (*53*). It is quite obvious that whey is produced in vast amounts and none of the current handling practices are effective enough for its exploitation or even valorisation (*54*).

Acid whey is rich in lactose (4.3%), proteins (0.5%), minerals (mainly calcium at 0.09%) and lactic acid (0.7%). The presence of lactic acid is the major drawback for valorisation of this byproduct stream, negatively affecting lactose crystallization and at the same time acting as an antimicrobial agent for the fermentation of whey into value-added products. Membrane separation has been proposed in the literature as a promising unit operation for the removal of lactic acid from acid whey (*51*, *55*). Chandrapala *et al.* (*51*) coupled nanofiltration with diafiltration and removed almost 66% lactic acid from acid whey. Ultrafiltration followed by electrodialysis led to 90% demineralization of acid whey, corresponding to almost 80% lactic acid removal in the study of Chen *et al.* (*55*).

**Fig. 1:**
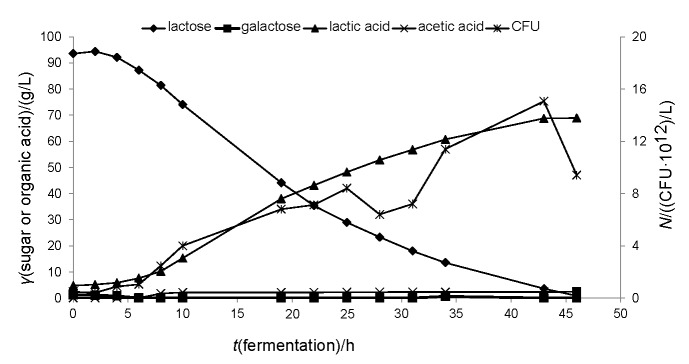
Sugar consumption, organic acid production and microbial growth in batch fermentation on technical scale (72-litre BIOSTAT UD bioreactor; B-Braun Biotech, Melsungen, Germany) using acid whey as feedstock, *V*=50 L. *L*. *coryniformis* subsp. *torquens* was used as the microbial biocatalyst. Fermentation was carried out at 30 °C and 200 rpm, and pH was adjusted to 6.0 by the addition of 20% (*m/m*) NaOH. Inoculum volume was 6% (*V/V*)

For this case study, acid whey (Glanbia Ingredients Ireland, Ballyragget, Ireland) was also nanofiltered in order to achieve higher lactose and at the same time lower lactic acid concentrations in the fermentation medium. The details of the nanofiltration will not be presented here, since the emphasis is given on the lactic acid fermentation. After nanofiltration, acid whey contained almost 100 g/L lactose and a small amount of galactose (1.2 g/L), while initial lactic acid concentration was 1.2 g/L. Yeast extract and MRS salts (in g/L: K_2_HPO_4_ 2, MgSO_4_ 0.1 and MnSO_4_ 0.05) were also added to the medium to support the growth of *Lactobacillus coryniformis* subsp. *torquens* (DSM 20005; DSMZ – German Collection of Microorganisms and Cell Cultures, Leibniz Institute, Braunschweig, Germany). Sugar consumption, organic acid formation and the number of viable cells expressed as colony forming units (CFU) per L are presented in Fig. 1. *L. coryniformis* subsp. *torquens* is an interesting strain that has not yet been fully studied in the literature. Even though it is classified as facultative heterofermentative, many researchers have reported its ability to produce d-lactate exclusively (*56*, *57*). Studies of the strain *L. coryniformis* subsp. *torquens* DSM 20004^T^ have shown that the strain can also produce acetate either under glucose limitation, or due to the presence of pentoses in the substrate (*57*).

In this experiment, acetic acid production was also observed, mainly during exponential growth phase (6–10 h), and its formation almost ceased during stationary phase. The strain was able to utilize the lactose present in the substrate, eliminating the need for prior hydrolysis. Fermentation lasted 46 h and 64.2 g/L of optically pure (99%) d-lactic acid was produced with a yield of 0.78 g/g based on total sugars and an average productivity of 1.40 g/(L·h). The final concentration of acetic acid was 2.4 g/L, while less than 1 g/L of lactose was left unconsumed. Juodeikiene *et al*. (*52*) tested d- and l-lactic acid production from hydrolysed cheese whey by employing the strains *L. bulgaricus* and *P. acidilactici*, respectively. The strain *L. bulgaricus* produced almost 57 g/L of optically pure d-lactic acid after 48 h of fermentation. The authors highlighted the use of CaCO_3_ as neutralizing agent in these experiments that is supposed to decrease the inhibitory effect of the produced lactic acid towards the microorganism. In a recent study of Liu *et al.* (*32*), cheese whey powder was hydrolysed using commercial proteases and utilized as a sole substrate for d-lactic acid production with the bacterial strain *L. bulgaricus* CGMCC 1.6970. Final d-lactic acid concentrations reached 70.7 and 113.2 g/L in batch and fed-batch mode, respectively. These results indicate that different waste streams from the dairy industry could be exploited for the production of d-lactic acid of high purity.

### Coffee mucilage

**Fig. 2:**
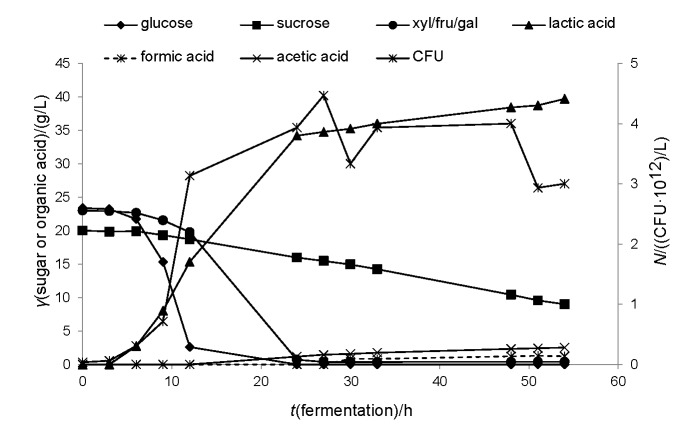
Sugar consumption, organic acid production and microbial growth in batch fermentation (2-litre bioreactor; Sartorius AG, Göttingen, Germany) using acid coffee mucilage as feedstock, *V*=1 L. *L. coryniformis* subsp. *torquens* was used as the microbial biocatalyst. Temperature was set at 30 °C, stirring at 400 rpm and the pH was adjusted to 6.0 by the addition of 20% (*m/m*) NaOH. Inoculum volume was 10% (*V/V*)

Coffee mucilage consists of the inner mesocarp of the bean, which is removed either by natural fermentation of the bean inside the tanks or mechanically (*58*). This waste material has already been considered as a potential substrate for bioconversion, since it is rich in reducing sugars (accounting for 63% *m*/*m*), proteins and pectins (*59*). Neu *et al.* (*8*) reported the production of more than 40 g/L of l-lactic acid of high purity from mucilage supplemented with 5 g/L yeast extract using a *B. coagulans* isolate. The production of d-lactic acid from coffee mucilage has not yet been reported in the literature. As in the case study using acid whey, the strain *L. coryniformis* subsp. *torquens* was evaluated for its ability to grow and produce d-lactic acid of high optical purity. Coffee mucilage was supplied by Cenicafé, the National Coffee Research Center in Manizales, Colombia. The composition of the material was (in %): dry matter 83.3, cellulose 4.7, hemicellulose 2.7, lignin 2.9, total sugars 40.7 (glucose 9.5, xylose/fructose/galactose 13, sucrose 18.2) and total Kjeldahl nitrogen 1.5. Mucilage was supplemented with 5 g/L yeast extract and also MRS salts. At the beginning of the fermentation, the substrate contained 23.4 g/L of glucose, 20 g/L of sucrose and 23 g/L of other monosaccharides. Due to analytical restrictions, it is not possible to determine the presence of either fructose or galactose or xylose, since they elute at the same time during HPLC analysis. The results are presented in Fig. 2. The strain showed a clear preference for glucose, since it was completely depleted after around 12 h. The consumption rate of both sucrose and the residual monosaccharides was slower at the beginning of the fermentation, but after glucose consumption, the other monosaccharides were also rapidly fermented. d-lactic acid accumulation initiated after 6 h and at the end of the fermentation (54 h), it reached a value of 39.7 g/L, with an optical purity of 99%. The yield on total sugars was 0.89 g/g, and the productivity was 0.74 g/(L·h). The production of acetic acid started when glucose was completely depleted (24 h) and when the concentration of the residual monosaccharides (xylose/fructose/galactose) also became limiting, formic acid was also produced (30 h). These results are in accordance with previous studies in which formic and acetic acids were produced by homofermentative strains due to different pyruvate metabolism, triggered either by a specific carbon source limitation, or by the assimilation of other sugars rather than glucose (*57*). Yáñez *et al.* (*57*) reported the consumption of hemicellulosic sugars when cardboard hydrolysate was utilized as substrate in simultaneous saccharification and fermentation. However, hemicellulosic sugars consist of both pentoses (xylose, arabinose) and hexoses (galactose, mannose), meaning that there is not yet enough information that the strain is actually able to assimilate pentoses.

At the end of the process, around 9 g/L of sucrose were still left unconsumed as well as 0.4 g/L of monosaccharides. Final concentrations of formic and acetic acids were 1.3 and 2.5 g/L, respectively. Comparing these results to the ones obtained when acid whey was employed as fermentation substrate, lactic acid yield was 14% higher, even though its average productivity decreased by 47%. It seems that the strain was able to consume lactose faster than sucrose, and also sucrose consumption possibly induced formic acid production (Fig. 2). Since this strain has not yet been fully explored, more research should be carried out in order to elucidate the different pathways involved during mixed sugar fermentation.

### Rice husks

During rice milling, the main byproduct streams include the husks and the bran. The first step of puddy rice processing involves the removal of the outer layer of the seed, which is called the rice husk. The processing of 1 kg of paddy rice generates approx. 0.2–0.33 kg husks (*60*). In 2017, 506 million tonnes of rice were estimated to have been harvested, meaning that rice husk generation was more than 100 million tonnes (*53*). Rice husks are rich in silica (90% of its ash content), which can be recovered and utilized (*61*). Many studies also deal with the hydrolysis of the hemicellulose and cellulose fraction for the production of bioethanol (*60*, *62*, *63*). Different pretreatment and hydrolysis methods have been suggested in the literature for the production of fermentable sugars (*64*, *65*).

In this case study, the details of the hydrolysis will not be described since this is not within the scope of the current review. However, after adequate pretreatment and hydrolysis (provided by Green Sugar AG, Meißen, Germany), a sugar-rich hydrolysate with 42.7 g/L total sugars was produced. The main carbohydrate present was possibly xylose at 28 g/L, followed by glucose (11 g/L) and lower amounts of cellobiose (3.7 g/L). A *Leuconostoc* sp. isolate (strain A15, available at Leibniz Institute for Agricultural Engineering and Bioeconomy, Potsdam, Germany) was selected for d-lactic acid production from this lignocellulosic substrate. *Leuconostoc* sp. is well-known heterofermentative LAB found in dairy (*66*) and other fermented products (*67*), but it has not yet been tested on a renewable substrate.

**Fig. 3:**
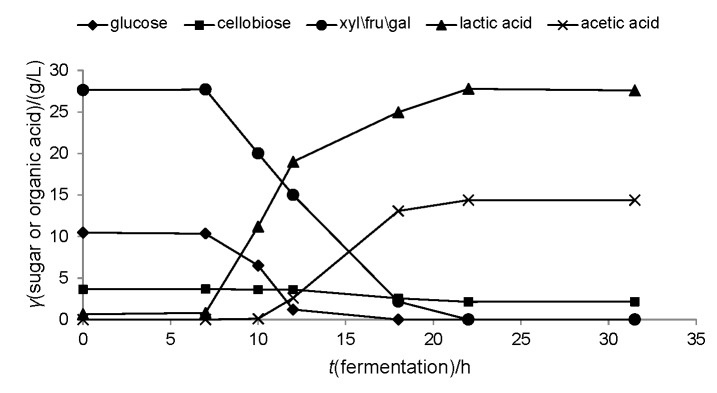
Sugar consumption and organic acid production in batch fermentation (2-litre bioreactor; Sartorius AG, Göttingen, Germany) using rice husk hydrolysate as feedstock. A *Leuconostoc* sp. isolate was used as the microbial biocatalyst. Temperature was set at 30 °C, stirring at 400 rpm and the pH was adjusted to 6.0 by the addition of 20% (*m/m*) NaOH. Inoculum volume was 10% (*V/V*)

The strain was able to utilize the sugars found in the hydrolysate, resulting in the production of approx. 27 g/L of d-lactic acid, with an optical purity of 97% (Fig. 3). As with the majority of strains, glucose was the first sugar to be consumed, but xylose was also depleted after 22 h of fermentation (Fig. 3). At this point the strain started to utilize cellobiose, but the consumption rate was too low and even after 49 h, only 1.4 g/L cellobiose was consumed. Lactic acid yield on total sugars was 0.74 g/g, and productivity was 0.88 g/(L·h). The main disadvantage of this process was that high concentrations of acetic acid were also formed (14.4 g/L). Further strain optimisation is required in order to achieve higher concentration of lactic acid compared to acetic acid.

## CONCLUSIONS

The biotechnological production of d-lactic acid from renewable resources is currently in the spotlight due to its significance in the production of polylactic acid. A wide variety of wild-type or genetically engineered LAB are able to produce high concentrations of optically pure d-lactic acid, but there is still need for research, especially in terms of utilization of lignocellulosic materials as fermentation feedstocks. Co-cultivation strategies could be an alternative for high d-lactic acid enantiomeric purity obtained *via* fermentation of mixtures of pentoses and hexoses. Genetically engineered strains could also be implemented, which will be able to produce lactic acid from hemicellulose following a homofermentative pathway. Our case studies showed that the strain *Lactobacillus coryniformis* subsp. *torquens* is an efficient d-lactic acid producer when cultivated in whey or in hydrolysates from coffee residues where no significant amounts of pentoses are present. *Leuconostoc* spp. is able to grow on lignocellulosic hydrolysates rich in pentoses, but further optimisation is required in order to minimize acetic acid formation.

## References

[1] Alves de Oliveira R Komesu A Vaz Rossell CE Maciel Filho R Challenges and opportunities in lactic acid bioprocess design – From economic to production aspects. Biochem Eng J. 2018;133:219–39. 10.1016/j.bej.2018.03.003

[2] Klotz S Kuenz A Prüße U Nutritional requirements and the impact of yeast extract on the d-lactic acid production by *Sporolactobacillus inulinus.* Green Chem. 2017;19:4633–41. 10.1039/C7GC01796K

[3] E4tech, RE-CORD, WUR. From the Sugar Platform to biofuels and biochemicals. Final Report for the European Commission, contract no. ENER/C2/423-2012/SI2.673791. European Commission; 2015. pp. 183. Available from: https://ec.europa.eu/energy/sites/ener/files/documents/EC%20Sugar%20Platform%20final%20report.pdf.

[4] Tsuji H Poly(lactide) stereocomplexes: Formation, structure, properties, degradation, and applications. Macromol Biosci. 2005;5(7):569–97. 10.1002/mabi.200500062 15997437

[5] Madhavan Nampoothiri K Nair NR John RP An overview of the recent developments in polylactide (PLA) research. Bioresour Technol. 2010;101(22):8493–501. 10.1016/j.biortech.2010.05.092 20630747

[6] Zhang J Tsuji H Noda I Ozaki Y Structural changes and crystallization dynamics of poly(l-lactide) during the cold-crystallization process investigated by infrared and two-dimensional infrared correlation spectroscopy. Macromolecules. 2004;37(17):6433–9. 10.1021/ma049288t

[7] Alexandri M Venus J Feedstock flexibility in sustainable chemistry: Bridging sectors still not sufficiently familiar with each other – Showcases of ongoing and emerging initiatives. Curr Opin Green Sustain Chem. 2017;8:24–9. 10.1016/j.cogsc.2017.09.003

[8] Neu AK Pleissner D Mehlmann K Schneider R Puerta-Quintero GI Venus J Fermentative utilization of coffee mucilage using *Bacillus coagulans* and investigation of down-stream processing of fermentation broth for optically pure l-(+)-lactic acid production. Bioresour Technol. 2016;211:398–405. 10.1016/j.biortech.2016.03.122 27035470

[9] Klotz S Kaufmann N Kuenz A Prüße U Biotechnological production of enantiomerically pure d-lactic acid. Appl Microbiol Biotechnol. 2016;100(22):9423–37. 10.1007/s00253-016-7843-7 27654657

[10] Awasthi D Wang L Rhee MS Wang Q Chauliac D Ingram LO Metabolic engineering of *Bacillus subtilis* for production of d-lactic acid. Biotechnol Bioeng. 2018;115(2):453–63. 10.1002/bit.26472 28986980

[11] Abdel-Rahman MA Tashiro Y Sonomoto K Recent advances in lactic acid production by microbial fermentation processes. Biotechnol Adv. 2013;31(6):877–902. 10.1016/j.biotechadv.2013.04.002 23624242

[12] Wang Y Tashiro Y Sonomoto K Fermentative production of lactic acid from renewable materials: Recent achievements, prospects, and limits. J Biosci Bioeng. 2015;119(1):10–8. 10.1016/j.jbiosc.2014.06.003 25077706

[13] Zhang Y Vadlani PV Kumar A Hardwidge PR Govind R Tanaka T Enhanced d-lactic acid production from renewable resources using engineered *Lactobacillus plantarum.* Appl Microbiol Biotechnol. 2016;100(1):279–88. 10.1007/s00253-015-7016-0 26433970

[14] Wang Q Ingram LO Shanmugam KT Evolution of d-lactate dehydrogenase activity from glycerol dehydrogenase and its utility for d-lactate production from lignocellulose. Proc Natl Acad Sci USA. 2011;108(47):18920–5. 10.1073/pnas.1111085108 22065761 PMC3223474

[15] Assavasirijinda N Ge D Yu B Xue Y Ma Y Efficient fermentative production of polymer-grade d-lactate by an engineered alkaliphilic *Bacillus* sp. strain under non-sterile conditions. Microb Cell Fact. 2016;15:3. 10.1186/s12934-015-0408-0 26754255 PMC4709905

[16] Liu Y Gao W Zhao X Wang J Garza E Manow R Pilot scale demonstration of d-Lactic acid fermentation facilitated by Ca(OH)_2_ using a metabolically engineered *Escherichia coli.* Bioresour Technol. 2014;169:559–65. 10.1016/j.biortech.2014.06.056 25103032

[17] Balakrishnan R Reddy Tadi SR Sivaprakasam S Rajaram S Optimization of acid and enzymatic hydrolysis of kodo millet (*Paspalum scrobiculatum*) bran residue to obtain fermentable sugars for the production of optically pure d (–) lactic acid. Ind Crops Prod. 2018;111:731–42. 10.1016/j.indcrop.2017.11.041

[18] Hama S Mizuno S Kihara M Tanaka T Ogino C Noda H Production of d-lactic acid from hardwood pulp by mechanical milling followed by simultaneous saccharification and fermentation using metabolically engineered *Lactobacillus plantarum.* Bioresour Technol. 2015;187:167–72. 10.1016/j.biortech.2015.03.106 25846187

[19] Okano K Hama S Kihara M Noda H Tanaka T Kondo A Production of optically pure d-lactic acid from brown rice using metabolically engineered *Lactobacillus plantarum.* Appl Microbiol Biotechnol. 2017;101(5):1869–75. 10.1007/s00253-016-7976-8 27832309

[20] Reddy Tadi SR Arun EVR Limaye AM Sivaprakasam S Enhanced production of optically pure d-(–)lactic acid from nutritionally rich *Borassus flabellifer* sugar and whey protein hydrolysate based–fermentation medium. Biotechnol Appl Biochem. 2017;64(2):279–89. 10.1002/bab.1470 26671214

[21] Wang X Wang G Yu X Chen H Sun Y Chen G Pretreatment of corn stover by solid acid for d-lactic acid fermentation. Bioresour Technol. 2017;239:490–5. 10.1016/j.biortech.2017.04.089 28549306

[22] Bai Z Gao Z Sun J Wu B He B d-lactic acid production by *Sporolactobacillus inulinus* YBS1-5 with simultaneous utilization of cottonseed meal and corncob residue. Bioresour Technol. 2016;207:346–52. 10.1016/j.biortech.2016.02.007 26897413

[23] Bai Z Gao Z He B Wu B Effect of lignocellulose-derived inhibitors on the growth and d-lactic acid production of *Sporolactobacillus inulinus* YBS1-5. Bioprocess Biosyst Eng. 2015;38(10):1993–2001. 10.1007/s00449-015-1440-5 26216317

[24] de Oliveira Moraes A Bojorge Ramirez NI Pereira Jr N Evaluation of the fermentation potential of pulp mill residue to produce d(−)-lactic acid by separate hydrolysis and fermentation using *Lactobacillus coryniformis* subsp. *torquens.* Appl Biochem Biotechnol. 2016;180:1574–85. 10.1007/s12010-016-2188-3 27424161

[25] Zhang Y Vadlani PV Lactic acid production from biomass-derived sugars *via* co-fermentation of *Lactobacillus brevis* and *Lactobacillus plantarum.* J Biosci Bioeng. 2015;119(6):694–9. 10.1016/j.jbiosc.2014.10.027 25561329

[26] Cui F Li Y Wan C Lactic acid production from corn stover using mixed cultures of *Lactobacillus rhamnosus* and *Lactobacillus brevis.* Bioresour Technol. 2011;102(2):1831–6. 10.1016/j.biortech.2010.09.063 20943382

[27] Prasad S Srikanth K Limaye AM Sivaprakasam S Homo-fermentative production of d-lactic acid by *Lactobacillus* sp. employing casein whey permeate as a raw feed-stock. Biotechnol Lett. 2014;36(6):1303–7. 10.1007/s10529-014-1482-9 24563313

[28] Yi X Zhang P Sun J Tu Y Gao Q Zhang J Engineering wild-type robust *Pediococcus acidilactici* strain for high titer l- and d-lactic acid production from corn stover feedstock. J Biotechnol. 2016;217:112–21. 10.1016/j.jbiotec.2015.11.014 26616423

[29] Zhao T Liu D Ren H Shi X Zhao N Chen Y d-lactic acid production by *Sporolactobacillus inulinus* Y2-8 immobilized in fibrous bed bioreactor using corn flour hydrolyzate. J Microbiol Biotechnol. 2014;24(12):1664–72. 10.4014/jmb.1406.06043 25085568

[30] Xu Q Zang Y Zhou J Liu P Li X Yong Q Highly efficient production of d-lactic acid from chicory-derived inulin by *Lactobacillus bulgaricus.* Bioprocess Biosyst Eng. 2016;39(11):1749–57. 10.1007/s00449-016-1650-5 27440161

[31] Beitel SM Sass DC Coelho LF Contiero J High D(-) lactic acid levels production by *Sporolactobacillus nakayamae* and an efficient purification. Ann Microbiol. 2016;66(4):1367–76. 10.1007/s13213-016-1224-4

[32] Liu P Zheng Z Xu Q Qian Z Liu J Ouyang J Valorization of dairy waste for enhanced d-lactic acid production at low cost. Process Biochem. 2018;71:18–22. 10.1016/j.procbio.2018.05.014

[33] Nguyen CM Kim JS Nguyen TN Kim SK Choi GJ Choi YH Production of l- and d-lactic acid from waste *Curcuma longa* biomass through simultaneous saccharification and cofermentation. Bioresour Technol. 2013;146:35–43. 10.1016/j.biortech.2013.07.035 23911815

[34] de la Torre I Ladero M Santos VE Production of d-lactic acid by *Lactobacillus delbrueckii* ssp. *delbrueckii* from orange peel waste: Techno-economical assessment of nitrogen sources. Appl Microbiol Biotechnol. 2018;102(24):10511–21. 10.1007/s00253-018-9432-4 30324487

[35] Cubas-Cano E González-Fernández C Ballesteros M Tomás-Pejó E Biotechnological advances in lactic acid production by lactic acid bacteria: Lignocellulose as novel substrate. Biofuels Bioprod Biorefin. 2018;12(2):290–303. 10.1002/bbb.1852

[36] Kango N Production of inulinase using tap roots of dandelion (*Taraxacum officinale*) by *Aspergillus niger.* J Food Eng. 2008;85(3):473–8. 10.1016/j.jfoodeng.2007.08.006

[37] Pleissner D Neu AK Mehlmann K Schneider R Puerta-Quintero GI Venus J Fermentative lactic acid production from coffee pulp hydrolysate using *Bacillus coagulans* at laboratory and pilot scales. Bioresour Technol. 2016;218:167–73. 10.1016/j.biortech.2016.06.078 27359065

[38] Liu G Sun J Zhang J Tu Y Bao J High titer l-lactic acid production from corn stover with minimum wastewater generation and techno-economic evaluation based on Aspen plus modeling. Bioresour Technol. 2015;198:803–10. 10.1016/j.biortech.2015.09.098 26454367

[39] López-Gómez JP Alexandri M Schneider R Venus J A review on the current developments in continuous lactic acid fermentations and case studies utilising inexpensive raw materials. Process Biochem. 2019;79:1–10. 10.1016/j.procbio.2018.12.012

[40] Palmqvist E Hahn-Hägerdal B Fermentation of lignocellulosic hydrolyzates. II: Inhibitors and mechanisms of inhibition. Bioresour Technol. 2000;74(1):25–33. 10.1016/S0960-8524(99)00161-3

[41] Zheng H Gong J Chen T Chen X Zhao X Strain improvement of *Sporolactobacillus inulinus* ATCC 15538 for acid tolerance and production of d-lactic acid by genome shuffling. Appl Microbiol Biotechnol. 2010;85(5):1541–9. 10.1007/s00253-009-2243-x 19777227

[42] Baek SH Kwon EY Kim YH Hahn JS Metabolic engineering and adaptive evolution for efficient production of d-lactic acid in *Saccharomyces cerevisiae.* Appl Microbiol Biotechnol. 2016;100(6):2737–48. 10.1007/s00253-015-7174-0 26596574

[43] Park HJ Bae J Ko H Lee S Sung HB Han J Low-pH production of d-lactic acid using newly isolated acid tolerant yeast *Pichia kudriavzevi*i NG7. Biotechnol Bioeng. 2018;115(9):2232–42. 10.1002/bit.26745 29896854

[44] Bae JH Kim HJ Kim MJ Sung BH Jeon JH Kim HS Direct fermentation of Jerusalem artichoke tuber powder for production of l-lactic acid and d-lactic acid by metabolically engineered *Kluyveromyces marxianus.* J Biotechnol. 2018;266:27–33. 10.1016/j.jbiotec.2017.12.001 29208409

[45] Jantasee S Kienberger M Mungma N Siebenhofer M Potential and assessment of lactic acid production and isolation – A review. J Chem Technol Biotechnol. 2017;92(12):2885–93. 10.1002/jctb.5237

[46] Kamble SP Barve PP Joshi JB Rahman I Kulkarni BD Purification of lactic acid *via* esterification of lactic acid using a packed column, followed by hydrolysis of methyl lactate using three continuously stirred tank reactors (CSTRs) in series: A continuous pilot plant study. Ind Eng Chem Res. 2012;51(4):1506–14. 10.1021/ie200642j

[47] Sanz MT Murga R Beltrán S Cabezas JL Coca J Kinetic study for the reactive system of lactic acid esterification with methanol: Methyl lactate hydrolysis reaction. Ind Eng Chem Res. 2004;43(9):2049–53. 10.1021/ie034031p

[48] Dimou C Vlysidis A Kopsahelis N Papanikolaou S Koutinas AA Kookos IK Techno-economic evaluation of wine lees refining for the production of value-added products. Biochem Eng J. 2016;116:157–65. 10.1016/j.bej.2016.09.004

[49] Gezae Daful A Görgens JF Techno-economic analysis and environmental impact assessment of lignocellulosic lactic acid production. Chem Eng Sci. 2017;162:53–65. 10.1016/j.ces.2016.12.054

[50] Sikder J Roy M Dey P Pal P Techno-economic analysis of a membrane-integrated bioreactor system for production of lactic acid from sugarcane juice. Biochem Eng J. 2012;63:81–7. 10.1016/j.bej.2011.11.004

[51] Chandrapala J Duke MC Gray SR Weeks M Palmer M Vasiljevic T Strategies for maximizing removal of lactic acid from acid whey – Addressing the un-processability issue. Separ Purif Tech. 2017;172:489–97. 10.1016/j.seppur.2016.09.004

[52] Juodeikiene G Zadeike D Bartkiene E Klupsaite D Application of acid tolerant *Pedioccocus* strains for increasing the sustainability of lactic acid production from cheese whey. Lebensm Wiss Technol. 2016;72:399–406. 10.1016/j.lwt.2016.05.023

[53] OECD. -FAO Agricultural Outlook 2018-2027. Rome, Italy: Food and Agriculture Organization of the United Nations; 2018. Available from: https://stats.oecd.org/viewhtml.aspx?QueryId=84948&vh=0000&vf=0&l&il=&lang=en#.

[54] Parashar A Jin Y Mason B Chae M Bressler DC Incorporation of whey permeate, a dairy effluent, in ethanol fermentation to provide a zero waste solution for the dairy industry. J Dairy Sci. 2016;99(3):1859–67. 10.3168/jds.2015-10059 26723112

[55] Chen GQ Eschbach FII Weeks M Gras SL Kentish SE Removal of lactic acid from acid whey using electrodialysis. Separ Purif Tech. 2016;158:230–7. 10.1016/j.seppur.2015.12.016

[56] Pinelli D González-Vara YRA Matteuzzi D Magelli F Assessment of kinetic models for the production of l- and d-lactic acid isomers by *Lactobacillus casei* DMS 20011 and *Lactobacillus coryniformis* DMS 20004 in continuous fermentation. J Ferment Bioeng. 1997;83(2):209–12. 10.1016/S0922-338X(97)83586-6

[57] Yáñez R Alonso JL Parajó JC d-lactic acid production from waste cardboard. J Chem Technol Biotechnol. 2005;80(1):76–84. 10.1002/jctb.1160

[58] Avallone S Guiraud JP Guyot B Olguin E Brillouet JM Polysaccharide constituents of coffee bean mucilage. J Food Sci. 2000;65(8):1308–11. 10.1111/j.1365-2621.2000.tb10602.x

[59] Orrego D Zapata-Zapata AD Kim D Optimization and scale-up of coffee mucilage fermentation for ethanol production. Energies. 2018;11(4):786 10.3390/en11040786

[60] Lim JS Abdul Manan Z Wan Alwi SR Hashim H A review on utilisation of biomass from rice industry as a source of renewable energy. Renew Sustain Energy Rev. 2012;16(5):3084–94. 10.1016/j.rser.2012.02.051

[61] Bakar RA Yahya R Gan SN Production of high purity amorphous silica from rice husk. Procedia Chem. 2016;19:189–95. 10.1016/j.proche.2016.03.092

[62] Ebrahimi M Villaflores OB Ordono EE Caparanga AR Effects of acidified aqueous glycerol and glycerol carbonate pretreatment of rice husk on the enzymatic digestibility, structural characteristics, and bioethanol production. Bioresour Technol. 2017;228:264–71. 10.1016/j.biortech.2016.12.106 28081524

[63] Wu J Elliston A Le Gall G Colquhoun IJ Collins SRA Wood IP Optimising conditions for bioethanol production from rice husk and rice straw: Effects of pre-treatment on liquor composition and fermentation inhibitors. Biotechnol Biofuels. 2018;11:62. 10.1186/s13068-018-1062-7 29541159 PMC5844111

[64] Wyman C, Decker S, Himmel M, Brady J, Skopec C, Viikari L. Hydrolysis of cellulose and hemicellulose. In: Severian D, editor. Polysaccharides: Structural diversity and functional versatility. New York, Marcel Dekker; 2004. pp.995–1033.

[65] Brodeur G Yau E Badal K Collier J Ramachandran KB Ramakrishnan S Chemical and physicochemical pretreatment of lignocellulosic biomass: A review. Enzyme Res. 2011;2011:787532. 10.4061/2011/787532 21687609 PMC3112529

[66] Kwon SY Garcia CV Song YC Lee SP GABA-enriched water dropwort produced by co-fermentation with *Leuconostoc mesenteroides* SM and *Lactobacillus plantarum* K154. Lebensm Wiss Technol. 2016;73:233–8. 10.1016/j.lwt.2016.06.002

[67] Xiong T Peng F Liu Y Deng Y Wang X Xie M Fermentation of Chinese sauerkraut in pure culture and binary co-culture with *Leuconostoc mesenteroides* and *Lactobacillus plantarum.* Lebensm Wiss Technol. 2014;59(2, Pt. 1):713–7. 10.1016/j.lwt.2014.05.059

